# Hepatitis E virus outbreak associated with rainfall in the Central African Republic in 2008-2009

**DOI:** 10.1186/s12879-020-04961-4

**Published:** 2020-04-03

**Authors:** Vianney Tricou, Julie Bouscaillou, Gina-Laure Laghoe-Nguembe, Aubin Béré, Xavier Konamna, Benjamin Sélékon, Emmanuel Nakouné, Mirdad Kazanji, Narcisse P. Komas

**Affiliations:** 1 Laboratoire des Arbovirus, Institut Pasteur de Bangui, Bangui, Central African Republic BP 923; 2 Institut Pasteur, Bangui, Central African Republic; 3 Laboratoire des Hépatites Virales, Institut Pasteur, Bangui, Central African Republic; 4 Laboratoire d’Analyses Médicales, Institut Pasteur, Bangui, Central African Republic; 5 Institut Pasteur, Bangui, Central African Republic, and Institut Pasteur, Cayenne, French Guiana

**Keywords:** Hepatitis E virus, Waterborne disease, Preventable disease, Epidemiology, Central Africa

## Abstract

**Background:**

Infection by hepatitis E virus (HEV) can cause a high burden of morbidity and mortality in countries with poor access to clean water and sanitation. Our study aimed to investigate the situation of HEV infections in the Central African Republic (CAR).

**Methods:**

A retrospective analysis of the blood samples and notification forms collected through the national yellow fever (YF) surveillance program, but for which a diagnosis of YF was discarded, was carried out using an anti-HEV IgM ELISA and a HEV-specific RT-PCR.

**Results:**

Of 2883 YF-negative samples collected between January 2008 and December 2012, 745 (~ 26%) tested positive by at least either of the 2 tests used to confirm HEV cases. The results revealed that the CAR was hit by a large HEV outbreak in 2008 and 2009. The results also showed a clear seasonal pattern with correlation between HEV incidence and rainfall in Bangui. A phylogenetic analysis showed that the circulating strains belonged to genotypes 1e and 2b.

**Conclusions:**

Overall, this study provides further evidences that HEV can be a significant cause of acute febrile jaundice, particularly among adults during rainy season or flood, in a country from Sub-Saharan Africa.

## Background

Hepatitis E virus (HEV) infection represents a significant public health problem in many parts of the world, particularly in Africa where it can cause outbreaks of acute hepatitis in areas with a high population density, heavy seasonal rainfall and high evapotranspiration rates [1, 2]. HEV has two epidemiological profiles in humans. HEV genotypes 3 and 4 typically cause sporadic cases in developed countries through zoonotic or foodborne transmission. In developing countries, HEV genotypes 1 and 2 are responsible for large waterborne outbreaks, which primarily occur among young adults during the rainy season. Symptomatic HEV infection consists in a short prodromal phase with asthenia, fever, abdominal pain, vomiting and anorexia that appears about 3 to 8 weeks after inoculation, followed by the appearance of jaundice [3]. While the disease is usually self-limited, outbreaks result in high attack rates in young adults and fatality rate among pregnant women can be up to 20% during the third trimester of pregnancy [4, 5]. While a marketed vaccine is available, the World Health Organization (WHO) does not recommend its routine use because of insufficient safety and efficacy data [6].

The Central African Republic (CAR) is among the poorest countries in the world. The CAR lacks basic infrastructures and access to essential sanitation, and it has endured decades of recurring violence and destructions. In 2015, the CAR ranked last in terms of Human Development Index [7]. HEV is endemic in the CAR with a reported seroprevalence up to 24% in young adults and regular outbreaks documented [8–11]. HEV infection represents an important differential diagnosis of yellow fever (YF) which is also endemic in the CAR and is closely monitored through a national program of surveillance. The aim of our work was to provide new insights into the HEV epidemiology and circulating strains in the CAR by using the data and samples collected between 2008 to 2012 through the YF surveillance.

## Methods

### Sample and data collection

We used longitudinal surveillance data from the national network of healthcare facilities, through which all suspected cases of YF were systematically captured. As recommended by the WHO, YF is suspected in case of acute onset of fever followed within 2 weeks by jaundice [12]. In case of suspected YF, healthcare facilities throughout the country, regardless of their level or sector, are asked to collect a blood sample, to fill out a standardized notification form (with date of jaundice onset, age, gender and province of residence), to place the sample in a cooler and to ship it immediately to the Institut Pasteur in Bangui (CAR’s capital and main city) for laboratory confirmation. All sera are then rapidly tested for the presence of IgM antibodies against YF virus by an IgM capture ELISA [13].

### Sample testing

Plasma samples collected between January 2008 and December 2012 through the YF surveillance and found negative for anti-YF IgM were retrospectively tested by HEV IgM ELISA Dia.Pro kit reference EVM.CE (Diagnostic Bioprobes srl, Milan, Italy) [14, 15] and/or by real-time RT-PCR, as previously described [16]. For the RT-PCR testing, viral RNA was extracted using QIAamp Viral RNA Mini Kit (QIAGEN, Courtaboeuf, France), and then retrotranscribed into cDNA using the High Capacity cDNA Reverse Transcription Kit (Applied Biosystems, Foster City, CA, USA). A HEV case was confirmed if the sample was positive for IgM antibodies and/or for viral RNA amplification.

### Phylogenetic analysis

A nested RT-PCR amplifying a 348-bp portion of the open reading frame 2 region was performed as previously described [17] on RNA samples selected amongst the samples that tested positive with the real-time RT-PCR. The amplicons were purified using QIAquick PCR Purification Kit (50), (QIAGEN, Hilden, Germany) and then sent to GATC Biotech (Konstanz, Germany) for direct sequencing. Phylogenetic analysis of the obtained sequences was performed using reference strains for different HEV genotypes and subtypes [18]. A maximum-likelihood tree was constructed using PhyML version 3.1 with 100 bootstrap replications and automatic substitution model selection by SMS [19, 20].

### Statistical analyses

Chi-square and Wilcoxon-Mann-Whitney tests were used to compare characteristics of HEV cases versus cases of other etiologies. Temporal and geographical distributions of HEV cases were descriptively analyzed. Incidence (provided as a rate per 100,000 persons per year) was calculated for each district using district population according to the last national census from 2003 [21]. Monthly occurrence of HEV cases in Bangui was compared with rainfall data from the Meteorology Unit of Bangui International Airport. Temporal correlations were examined statistically by computing the Pearson correlation coefficient. Year period from May to October was considered as the rainy season. Statistical analyses were performed using STATA 11.0.

## Results

### HEV testing results

**Fig. 1:**
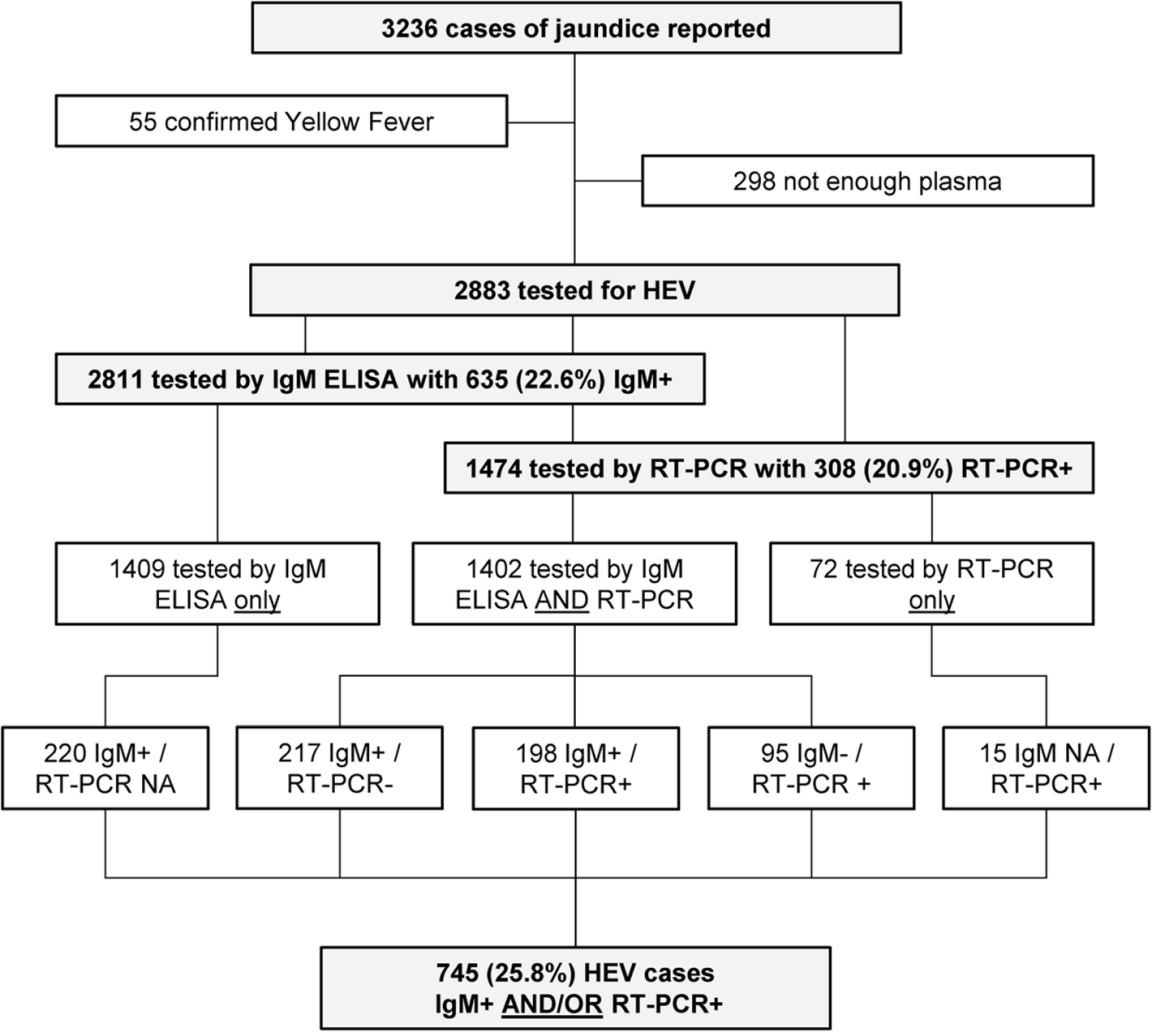
Flow chart of the HEV testing. NA: not available

Between January 2008 and December 2012, 3236 samples were collected and tested for YF-specific IgM at the Institut Pasteur in Bangui [13]. Of 3181 YF-negative samples, 2883 were tested for HEV-specific IgM and/or viral RNA (1402 [48.6%] were tested for both, 1409 for HEV IgM only [48.9%], and 72 [2.5%] for HEV RNA only), 298 had insufficient volume (Fig. 1). Of note, the proportion of samples that could be tested was different according to the year, ranging from 80.7% in 2008 to 97.7% in 2012 (Additional Table 1). Overall, 635 samples tested positive for HEV IgM (22.6% of the 2811 samples tested by ELISA), and 308 samples tested positive for HEV RNA (20.9% of the 1474 samples tested by real-time RT-PCR). When considering positivity for HEV IgM and/or viral RNA, 745 (25.8% of the 2883 samples tested by at least one test) were positive for HEV (Fig. 1).

### Characteristics of HEV cases

Among HEV cases, 44.0% were women, and the median age was 23 years. Compared to jaundice cases of other etiology, HEV cases were significantly associated with older age, urban setting and rainy season (Table 1). The proportion of HEV cases among jaundice cases varied significantly with age, ranging from ~ 14% in cases ≤ 5 years of age to ~ 30% in cases > 15 years old (*p* < 0.001) (Additional Table 2).

**Table 1:** Characteristics of the HEV cases compared to cases of other etiologies

	Total		HEV cases		Cases of other etiologies		*p*
	*N* = 2883		*N* = 745		*N* = 2138		
**Sex** n, %							
Female	1239	43.0%	327	44.0%	912	42.7%	0.445
Male	1642	57.0%	417	56.0%	1225	57.3%	
**Age** (median, IQR)	21, 11–30		23, 17–33		20, 8–30		< 0.001
**Year** n, %							
2008	501	17.4%	270	36.3%	231	10.8%	< 0.001
2009	813	28.2%	352	47.3%	461	21.6%	
2010	615	21.3%	83	11.2%	532	24.9%	
2011	438	15.2%	25	3.4%	413	19.3%	
2012	516	17.9%	15	2.0%	501	23.4%	
**Season** n, %							
Dry	1257	43.6%	254	34.1%	1003	46.9%	< 0.001
Rainy	1626	56.4%	491	65.9%	1135	53.1%	
**Location** n, %							
Bangui	1037	36.1%	356	47.8%	681	31.9%	< 0.001
Rest of the country	1839	63.9%	388	52.2%	1451	68.1%	

Gender was missing for 2 cases; location was missing for 7 cases

### Temporal and geographical distribution of HEV cases

**Fig. 2:**
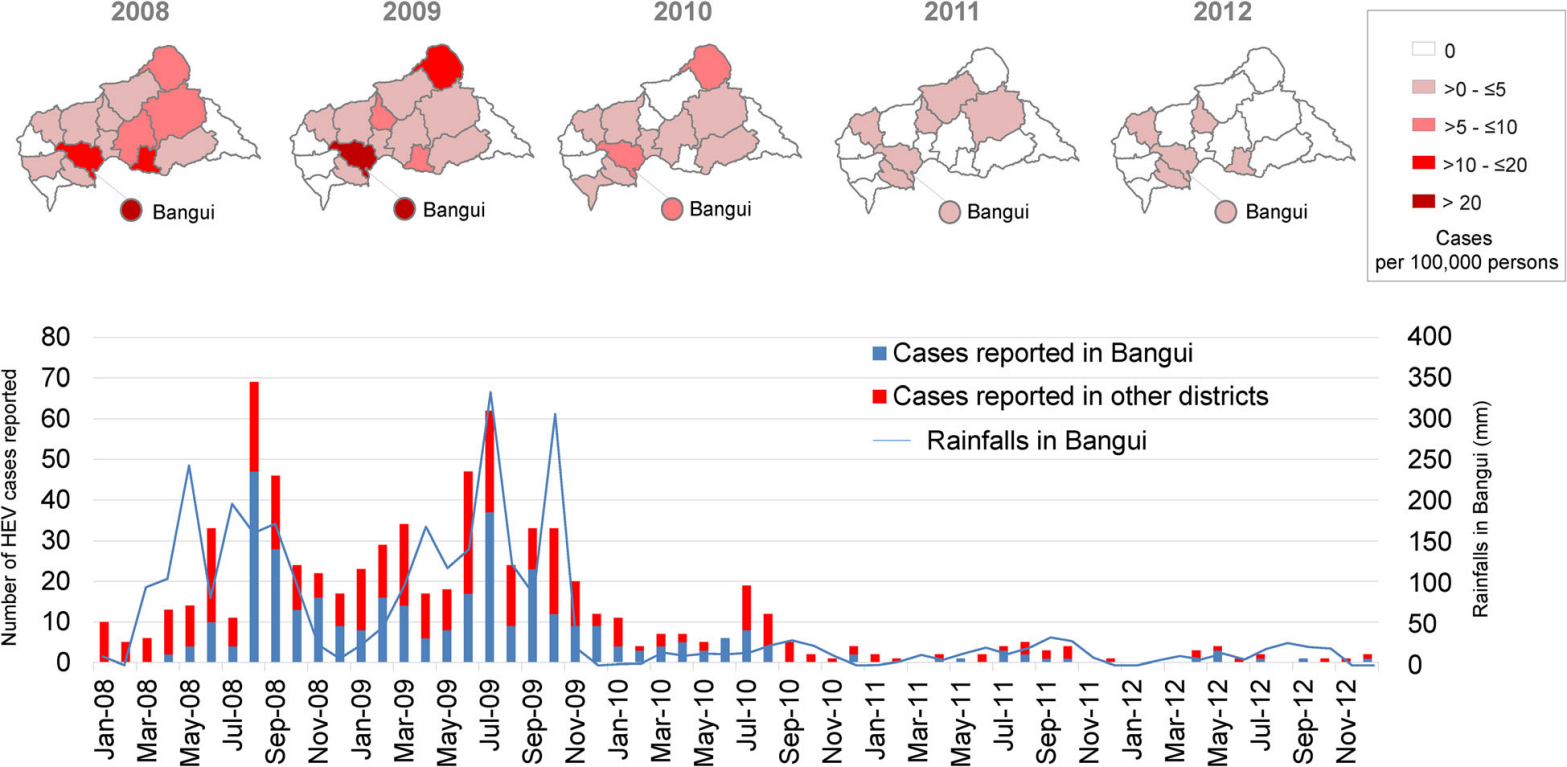
Temporal and geographical distribution of HEV cases in the CAR with the monthly rainfall in Bangui – 2008-2012

The monthly number of HEV cases showed a seasonal pattern, with an average peak incidence between June and September (Fig. 2). Geographical distribution by year highlighted a major epidemic situation across the whole country in 2008–2009 with up to 27 reported cases per 100,000 person-years in Bangui in 2009. In addition, the monthly number of HEV cases in Bangui was strongly correlated with considerable precipitation during the 2008 and 2009 rainy seasons (rho spearman 0.563, *p* < 0.001) (Fig. 2).

### Phylogenetic analysis

**Fig. 3:**
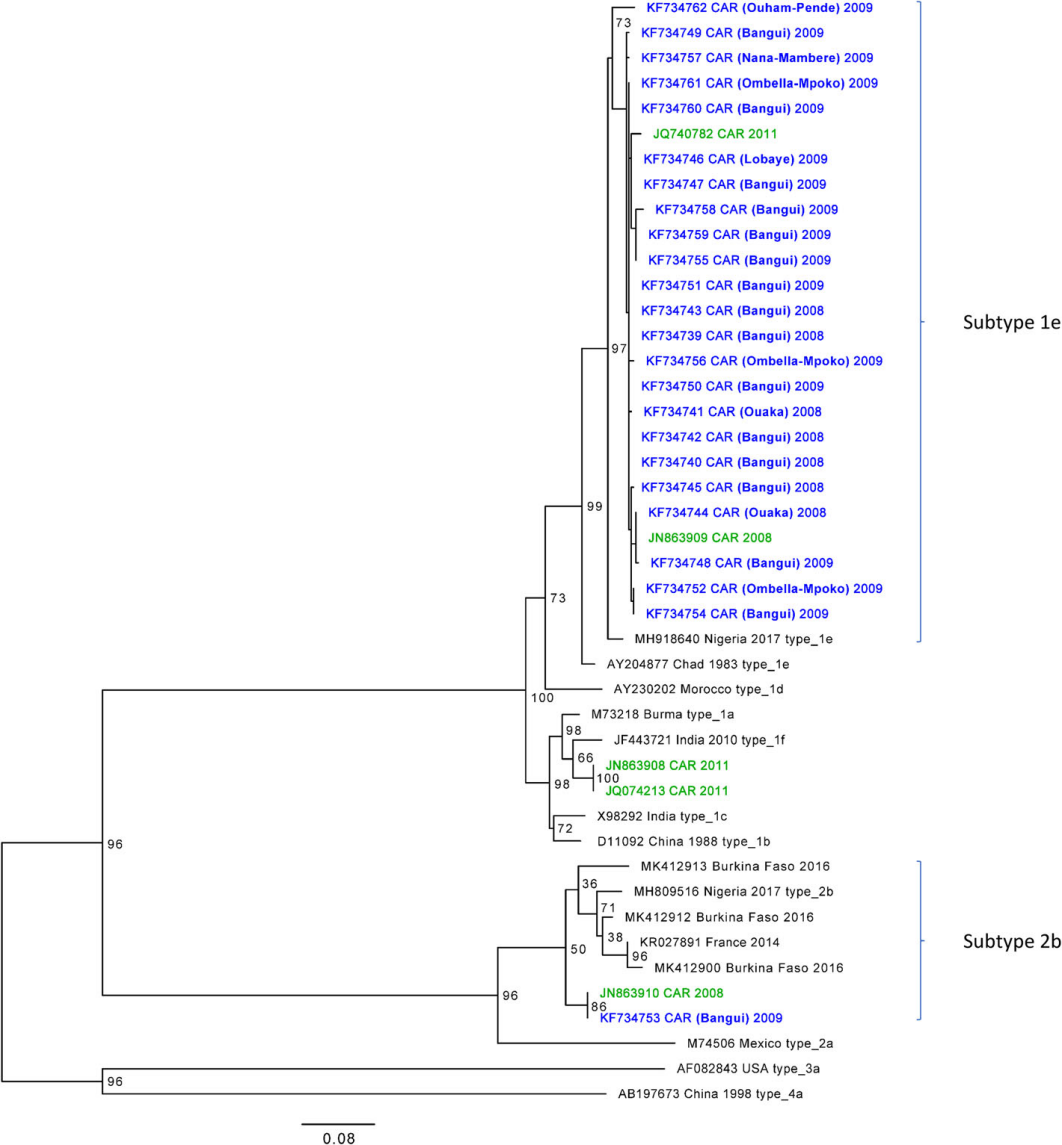
Phylogenetic analysis of HEV strains from the CAR – 2008-2012. Scale bar represents nucleotide substitutions per site. Percentages of replicate trees in which the associated virus isolates clustered together in the bootstrap test (100 replicates) are shown next to the branches. The sequences generated in this study are depicted in blue. The sequences generated in Bouscaillou et al. 2013 [11] are depicted in green. These sequences are also from samples collected in the CAR during the period 2008–2012

Ninety-six RNA samples were analyzed by the nested RT-PCR with 24 high quality sequences obtained (GenBank accession numbers from KF734739 to KF734762). Phylogenetic analysis of these sequences showed closely related viruses belonging to genotype 1 subtype e in 23 samples (~ 93–100% of nucleotide sequence identity) (Fig. 3). These viruses are closely related to strains from other sub-Saharan African countries with up to ~ 95% of nucleotide sequence identity (Fig. 3) [22]. The phylogenetic analysis also showed one strain that belongs to genotype 2 subtype b that is closely related (~ 90–93% of identity) to strains isolated from an outbreak in Nigeria in 2017 [23], in France from a person with history of recent travel to Senegal [24], and during a recent outbreak in Burkina Faso (Fig. 3) [25]. Of note, this strain is highly related to the strain isolated in 2008 in the CAR (100% of identity) [11].

## Discussion

We report here the use of a vast collection of samples and related notification forms that represented a unique opportunity to investigate the epidemiology of HEV infection in the CAR. These country-level five-year results confirm that HEV can be a significant cause of acute febrile jaundice in the CAR, particularly in adults during the rainy season or following flood. Therefore, a diagnosis of HEV should be considered in case of clustered febrile jaundice cases, i.e. ≥5 cases sharing the same water supply [26]. The addition to the notification form used by the YF surveillance of a question about the water supply of the icteric case should also be considered. This could contribute to the early identification of contaminated water supplies and the rapid implementation of corrective actions. Given the current situation in the CAR with recurrent population displacements and destruction, our data also strongly supports the implementation of preventive actions like the development of outbreak response plans for high-risk areas (camp of refugees and internally-displaced people) that include elements about early diagnosis.

These results showed a large outbreak across the CAR in 2008–2009 particularly in Bangui, which is the capital city and main urban center of the country (Bangui concentrates about 1/5 of the country population). Overall, there was about a quarter of the febrile jaundice cases collected through the YF surveillance being tested positive for HEV. Differences in seroprevalence between rural and urban areas have been reported. In Gabon, one study found a higher prevalence in urban compared with rural areas, while another study in South Africa found a higher HEV seroprevalence in rural compared with urban areas [27, 28]. In our study (which was not a seroprevalence study), a higher incidence (34% versus 21% of the febrile jaundice cases) was found in Bangui compared to the rest of the country. This might be due to the higher population density in Bangui with water supplies that are shared by a lot of people [2]. However, the incidence in the rest of the country was far from negligible with detected strains that are closely related to strains isolated in Bangui. This suggests that the circulation of HEV in the CAR was highly dynamic, and both urban and rural areas had poor quality water sources that spread these pathogens.

Results from the phylogenetic analysis are consistent with previous findings regarding the subtypes circulating in the CAR and the neighboring countries, and with waterborne transmission usually associated with HEV genotypes 1 and 2 [9, 11]. The high number of cases observed in Bangui in 2008–2009 following heavy rains suggests that large water sources could have been contaminated with fecal matters. Isolated cases could be due to contamination of limited water sources. Chains of asymptomatic infections of young children with adult relatives already immune might explain recurrent outbreaks. Prolonged viral excretion may also account for recurrent outbreaks.

However, our study had several limitations. Our results might highly underestimate incidence of HEV infections, as asymptomatic infections were not captured. In addition, not all samples were tested for HEV, nor were all samples that were tested for IgM also tested for viral RNA because of insufficient volume of some samples. In addition, 72 samples were tested by RT-PCR only because of shortage of the HEV IgM ELISA kit in the laboratory at the time of the testing, but with a likely limited impact on the study outcome given the low number of samples. It is also possible that the YF surveillance nationwide coverage was incomplete.

## Conclusions

This study provides further evidences that HEV can be a leading cause of febrile jaundice cases in a Sub-Saharan country, particularly in the adults during the rainy season or following flood. These data also highlight the urgency of generating additional data to support the use of recently marketed HEV vaccine to mitigate or prevent outbreaks of hepatitis E and its consequences in high risk groups [29, 30]. Finally, our study also provides an example of how to use YF national surveillance program data to investigate another disease with a significant impact on populations.

## Supplementary information

**Additional file 1: Table S1.** Number of positive samples for HEV by year of sampling and by test.

**Additional file 2 : Table S2.** Distribution of HEV cases by age groups and by test.

## Data Availability

The sequences generated in this study are available in GenBank under accession numbers KF734739 – KF734762. Data of the phylogenetic analysis are available at TreeBase with accession number S25902. The de-identified clinical dataset generated and analyzed during this study is available upon request from the corresponding author.

## Abbreviations

CAR: Central African Republic
HEV: Hepatitis E virus
IgM: Immunoglobulin M
RNA: Ribonucleic acid
RT-PCR: Reverse transcriptase - polymerase chain reaction
YF: Yellow fever
WHO: World Health Organization
