# Supplementary material for: Hepatitis E virus outbreak associated with rainfall in the Central African Republic in 2008-2009
Source: BMC Infect Dis. 2020 Apr 3;20:260. doi: 10.1186/s12879-020-04961-4 (PMC7119096; doi:10.1186/s12879-020-04961-4)
Supplement: Supplementary file 1 — Additional file 1: Table S1. Number of positive samples for HEV by year of sampling and by test. [file 12879_2020_4961_MOESM1_ESM.docx]

**Additional Table 1: Number of positive samples for HEV by year of sampling and by test.**

| Year of sampling | Samples negative for YF | Samples tested for HEV | Samples tested by IgM ELISA and  RT-PCR | Samples IgM+ /  RT-PCR- | Samples IgM+ /  RT-PCR+ | Samples IgM- /  RT-PCR+ | Samples tested by IgM ELISA only | Samples IgM+ /  RT-PCR NA | Samples tested by RT-PCR only | Samples IgM NA /  RT-PCR+ |
| --- | --- | --- | --- | --- | --- | --- | --- | --- | --- | --- |
| 2008 | 621 | 501 (80.7%) | 309 | 68 (22.0%) | 88 (28.5%) | 20 (6.5%) | 185 | 92 (49.7%) | 7 | 2 (28.6%) |
| 2009 | 852 | 813 (95.4%) | 517 | 120 (23.2%) | 75 (14.5%) | 61 (11.8%) | 246 | 83 (33.7%) | 50 | 13 (26.0%) |
| 2010 | 722 | 615 (85.2%) | 442 | 16 (3.6%) | 33 (7.5%) | 12 (2.7%) | 162 | 22 (13.6%) | 11 | 0 |
| 2011 | 458 | 438 (95.6%) | 116 | 2 (1.7%) | 0 | 0 | 318 | 23 (7.2%) | 4 | 0 |
| 2012 | 528 | 516 (97.7%) | 18 | 11 (61.1%) | 2 (11.1%) | 2 (11.1%) | 498 | 0 (0.0%) | 0 | 0 |
| Total | 3181 | 2883 (90.6%) | 1402 | 217 (15.5%) | 198 (14.1%) | 95 (6.8%) | 1409 | 220 (15.6%) | 72 | 15 (20.8%) |

NA: not available
