# Supplementary material for: Hepatitis E virus outbreak associated with rainfall in the Central African Republic in 2008-2009
Source: BMC Infect Dis. 2020 Apr 3;20:260. doi: 10.1186/s12879-020-04961-4 (PMC7119096; doi:10.1186/s12879-020-04961-4)
Supplement: Supplementary file 2 — Additional file 2 : Table S2. Distribution of HEV cases by age groups and by test. [file 12879_2020_4961_MOESM2_ESM.docx]

**Additional Table 2: Distribution of HEV cases by age groups and by test.**

| Age group | Samples tested for HEV | Samples IgM+ and/or  RT-PCR+ | Samples tested by IgM ELISA | Samples IgM+ | Samples tested by RT-PCR | Samples PCR+ |
| --- | --- | --- | --- | --- | --- | --- |
| ≤5 year | 477 | 66 (13.8%) | 464 | 45 (9.7%) | 248 | 29 (11.7%) |
| 6-15 | 489 | 97 (19.8%) | 478 | 71 (14.9%) | 244 | 54 (22.1%) |
| 16-25 | 840 | 260 (31.0%) | 820 | 235 (28.7%) | 417 | 109 (26.1%) |
| 26-35 | 555 | 167 (30.1%) | 540 | 148 (27.4%) | 298 | 64 (21.5%) |
| 36-45 | 275 | 80 (29.1%) | 268 | 73 (27.2%) | 144 | 28 (19.4%) |
| 46-55 | 149 | 48 (32.2%) | 146 | 42 (28.8%) | 72 | 13 (18.1%) |
| 56+ | 98 | 27 (27.6%) | 95 | 21 (22.1%) | 51 | 11 (21.6%) |
| Total | 2883 | 745 (25.8%) | 2811 | 635 (22.6%) | 1474 | 308 (20.9%) |
